# Pharmacovigilance Pregnancy Data in a Population of Japanese Patients With Chronic Inflammatory Disease Exposed to Certolizumab Pegol

**DOI:** 10.1111/1756-185X.70048

**Published:** 2025-01-15

**Authors:** Mikako Goto, Shigeru Saito, Angela E. Scheuerle, Shinya Yasuda, Niamh Houston, Thomas Kumke, Bernard Lauwerys, Atsuko Murashima

**Affiliations:** ^1^ Japan Drug Information Institute in Pregnancy National Center for Child Health and Development Tokyo Japan; ^2^ University of Toyama Toyama Japan; ^3^ UT Southwestern Medical Center Dallas Texas USA; ^4^ UCB Tokyo Japan; ^5^ UCB Slough UK; ^6^ UCB Monheim am Rhein Germany; ^7^ UCB Brussels Belgium

**Keywords:** Certolizumab pegol, congenital anomalies, Japanese patients, pregnancy outcomes, TNFi

## Abstract

**Aim:**

Uncontrolled chronic inflammatory diseases (CIDs) before, during, and after pregnancy, as well as some CID medications, can increase the risk of impaired fertility in addition to adverse maternal/pregnancy outcomes in women of childbearing age. We report pregnancy outcomes from prospectively reported pregnancies in Japanese women treated with certolizumab pegol (CZP).

**Methods:**

Data from July 2001 to November 2020 on CZP‐exposed pregnancies from the CZP Pharmacovigilance safety database were reviewed. Pregnancy outcomes analyzed included live birth, ectopic pregnancy, abortion (miscarriage or medically indicated/elective), and stillbirth. Congenital anomalies (major and minor), preterm delivery, and low birth weight were also examined.

**Results:**

Among 149 prospective pregnancies with maternal CZP exposure and known outcomes identified in Japanese women, 111/149 (74.5%) involved at least first‐trimester exposure and 53/149 (35.6%) were exposed in all trimesters; 135/149 (90.6%) live births, 12/149 (8.1%) abortions (11 miscarriages, one elective termination), 2/149 (1.3%) stillbirths, no ectopic pregnancies reported. One (0.7%) infant, whose mother had first‐trimester exposure, manifested a minor congenital anomaly (accessory auricle). There were no major congenital anomalies. Among live births, 3/135 (2.2%) were preterm and 10/135 (7.4%) had low birth weight.

**Conclusion:**

The safety profile of CZP in pregnant Japanese women was consistent with published global data.

## 1 Introduction

Women of childbearing age are vulnerable to chronic inflammatory diseases (CIDs) such as axial spondyloarthritis (axSpA), psoriatic arthritis (PsA), psoriasis (PSO), and rheumatoid arthritis (RA), some of which typically manifest in early adulthood and therefore tend to overlap with their peak reproductive years [1, 2, 3, 4, 5]. Uncontrolled CID activity before, during, and after pregnancy has been reported to be associated with increased risk of impaired fertility and adverse maternal outcomes, as well as adverse outcomes in pregnancy, including miscarriage, preterm delivery, and low birth weight [6, 7, 8, 9, 10]. Pharmacological interventions which provide adequate disease control before, during, and after pregnancy would therefore be highly desirable to patients with CID who are pregnant or planning pregnancy. The pursuit of such treatments is strongly aligned with international guidelines on the care of women of childbearing age with CIDs, including those of the American College of Rheumatology (ACR), British Society for Rheumatology (BSR), and European Alliance of Associations for Rheumatology (EULAR) [11, 12, 13].

Although conventional therapies including corticosteroids and non‐steroidal anti‐inflammatory drugs (NSAIDs) are available, prior publications have reported potential links between these drugs and fertility impairment as well as adverse pregnancy outcomes [9, 14]. Conventional synthetic disease‐modifying anti‐rheumatic drugs (csDMARDs) such as methotrexate are also commonly used for treating CIDs including RA and PsA [15]. However, methotrexate has well‐established risks of teratogenic effects and is therefore contraindicated in pregnancy [15]. Current guidelines on the treatment of CIDs in pregnant women state that tumor necrosis factor inhibitor (TNFi) use may be considered if clinically required [11, 12, 13]. However, some TNFi agents are recommended for discontinuation in the second or third trimester unless there is a disease flare [11, 12]. Discontinuation serves as a precautionary measure against placental transfer of the drugs and in utero fetal exposure in the second and third trimesters which may heighten the risk for infections in infants [16, 17]. Another notable consequence of using TNFi biologics in pregnant women is the limitation on postpartum administration of live vaccines in infants. According to current recommendations, attenuated live vaccinations should be avoided for a minimum of 6 months after birth in infants of mothers treated with TNFi therapeutics during the second half of pregnancy [13, 17, 18, 19]; in the case of infliximab, at least 12 months postpartum [20]. These recommendations were established following a case report of infant death due to disseminated Bacillus Calmette‐Guérin (BCG) after vaccination at 3 months of age [21]. This was hypothesized to be associated with in utero infliximab exposure throughout pregnancy [21]. Further cases of fatal disseminated BCG infections in TNFi‐exposed infants have since been reported [22], underscoring the need for therapies which minimize fetal TNFi exposure.

Certolizumab pegol (CZP) is a PEGylated, Fc‐free TNFi approved in Japan for the treatment of erythrodermic psoriasis, generalized pustular psoriasis, PsA, PSO, and RA [23, 24]. According to guidance from Japan's Pharmaceuticals and Medical Devices Agency, CZP may be administered to pregnant women or women who may be pregnant if the therapeutic benefit outweighs the risk [24]. Unlike other TNFi biologics, CZP lacks an immunoglobulin G (IgG) Fc portion and therefore does not bind the neonatal Fc receptor from the first trimester onward, preventing the antibody from crossing the placental barrier from mother to fetus by active transfer [25, 26]. Transfer by passive diffusion, if any, is known to be minimal [19]. A previously published pharmacokinetic study of CZP in pregnant women indicated low to minimal in utero fetal exposure during the third trimester based on maternal and infant CZP plasma concentrations at delivery and post‐delivery (infants only). These findings supported the continued use of the drug throughout pregnancy in women with CIDs where clinically needed [11, 23]. Additionally, the ACR, BSR, and EULAR guidelines state that CZP is compatible with all trimesters of pregnancy in women of childbearing age who also plan to use TNFi drugs [11, 12, 13].

Pregnancy outcomes in 1392 prospective pregnancies with known outcomes and maternal CZP exposure from the CZP Pharmacovigilance safety database were previously published [27]. Among these pregnancies, no increased risk of adverse outcomes or numbers and patterns of congenital anomalies were reported when compared with the general population [27, 28]. Evidence on pregnancy outcomes in the TNFi‐exposed Japanese population, however, is currently lacking. New safety data from a pharmacovigilance database would thus be valuable to healthcare professionals and women of childbearing age in enabling informed benefit–risk assessments and individual decision‐making concerning CZP treatment during pregnancy.

Here, we present data from prospectively reported pregnancies with known outcomes in Japanese women treated with CZP from the CZP Pharmacovigilance safety database.

## 2 Materials and Methods

### 2.1 Data Source and Patient Populations

This post hoc analysis of Japanese patients treated with CZP was conducted in May 2022, and utilized the methodology reported in a previous global study [27]. Briefly, pregnancy data from the CZP Pharmacovigilance safety database global cohort were reviewed from the start of CZP clinical development (earliest report July 12, 2001) up to the cut‐off date of November 1, 2020. These data were consolidated from spontaneous reports (obtained directly from patients or healthcare providers), published literature, as well as interventional and non‐interventional studies. Definitions of safety outcomes from the global study were also applied to this Japanese analysis.

This analysis focused on prospectively reported pregnancies with known outcomes in Japanese women to minimize potential reporting bias associated with retrospective pregnancy cases. Similar to the global study, a prospectively reported pregnancy was defined as one in which CZP exposure was reported prior to the knowledge of the pregnancy outcome or prior to detection of a congenital anomaly at prenatal examination [27, 28]. In contrast, retrospective pregnancies were defined as CZP‐exposed pregnancies reported after the pregnancy outcomes were known or after a fetal abnormality was detected by prenatal testing [27, 28].

### 2.2 Data Collected

#### 2.2.1 Maternal Demographics and Baseline Characteristics

Timing of maternal CZP exposure was categorized as during preconception only (up to 70 days prior to conception), at least the first trimester, during all trimesters, or otherwise (second trimester only, third trimester only, or both). The first trimester was defined as the period up to 13 weeks and 6 days' gestation, the second as 14–27 weeks and 6 days' gestation, and the third as any time at or after 28 weeks' gestation. Pregnancies without information on timing of CZP exposure were reported as “missing.”

Information on maternal characteristics obtained from the database included indication(s) for CZP treatment, type of birth (singleton or others), maternal age at the date of conception, obstetric complications (gestational diabetes, hypertensive disease of pregnancy and maternal infections), and concomitant medications for CID.

#### 2.2.2 Pregnancy Outcomes

Pregnancy outcomes were descriptively reported and categorized according to live birth, ectopic pregnancy, abortion (miscarriage or induced termination [medically indicated or elective]), and stillbirth. In the global study, miscarriage was defined as non‐induced embryonic/fetal death before 20 weeks of gestation and stillbirth was defined as fetal death occurring at or after 20 weeks of gestation [27]. The same definitions were applied in this Japanese analysis. Additional outcomes assessed for live birth cases include congenital anomalies, preterm delivery (born alive before 37 weeks of pregnancy), and low birth weight (less than 2500 g) [29, 30]. Pregnancies with unknown outcomes, which comprised ongoing pregnancies and those with no reported pregnancy outcomes, were excluded from this analysis.

Cases of congenital anomalies were assessed by an external teratologist and classified as major or minor based on the Metropolitan Atlanta Congenital Defects Program (MACDP) and the European Surveillance of Congenital Anomalies (EUROCAT) classification criteria [31, 32].

Data on pregnancy outcomes, including congenital anomalies, from the global study are also reported here to contextualize the findings of this Japanese analysis.

### 2.3 Statistical Analysis

Descriptive statistics were used to summarize the data. Means and standard deviations were calculated for continuous maternal baseline characteristics. For binary maternal baseline characteristics and pregnancy outcomes, the number of cases and proportions were calculated. No statistical tests were conducted.

## 3 Results

### 3.1 Patient Disposition and Baseline Characteristics

**FIGURE 1:**
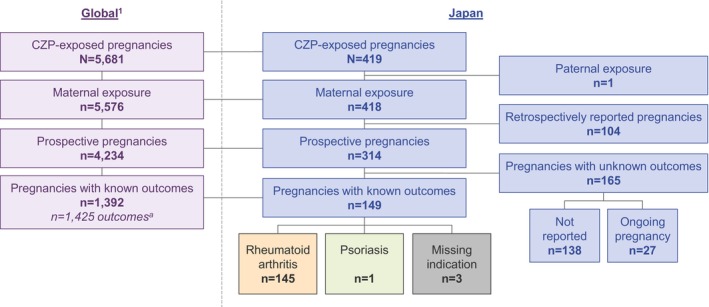
Reports of pregnancies with CZP exposure identified in the CZP Pharmacovigilance safety database. ^a^The higher number of outcomes compared with pregnancies in the global population is due to a number of non‐singleton births. 1. Clowse M, et al. Ther Adv Musculoskel Dis 2022;14:1–18. CZP: Certolizumab pegol.

Up to the November 1, 2020 cut‐off date, 419 reports of CZP‐exposed pregnancies in Japanese patients were identified in the CZP Pharmacovigilance safety database, of which 418/419 (99.8%) involved maternal exposure (Figure 1). Of these Japanese women, known outcomes were recorded in 149 prospective pregnancies. Indications for CZP treatment which were identified consisted of RA (*n* = 145) and PSO (*n* = 1); three pregnancies had missing diagnosis data (Figure 1).

### 3.2 Prospective Pregnancies With Known Outcomes

#### 3.2.1 Maternal Demographics and Baseline Characteristics

Most (117/149; 78.5%) prospective pregnancy records with maternal CZP exposure and known outcomes were obtained from spontaneous and literature reports, whereas the rest were from clinical trials (3/149; 2.0%), or non‐interventional sources (29/149; 19.5%) (Table 1).

**TABLE 1:** Demographics and baseline characteristics of prospective pregnancies with known pregnancy outcomes and maternal CZP exposure.

	Pregnancies with known outcomes	
	Japan	Global^a^
Number of pregnancies	149	1392
Number of outcomes	149	1425^b^
Report source, *n* (%)^c^		
NIS/Registries/Other	29 (19.5)	724 (52.0)
Spontaneous reports	117 (78.5)	577 (41.5)
Clinical trials	3 (2.0)	91 (6.5)
Type of birth, *n* (%)^c^		
Singleton	149 (100)	1360 (97.7)
Others	0	32 (2.3)
Maternal age, years, mean (SD)	34.3 (5.3)	31.9 (5.1)
Maternal age categories, years		
< 18	0	2 (0.1)
≥ 18 to ≤ 35	76 (51.0)	883 (63.4)
> 35	60 (40.3)	305 (21.9)
Missing	13 (8.7)	202 (14.5)
CZP exposure, *n* (%)^c^		
Preconception only	1 (0.7)	17 (1.2)
At least first trimester^d^	111 (74.5)	1021 (73.3)
All trimesters	53 (35.6)	547 (39.3)
Other^e^	37 (24.8)	313 (22.5)
Missing	0	41 (2.9)
Obstetric complications		
Gestational diabetes	0	37 (2.7)
Hypertensive disease of pregnancy^f^, ^g^	2 (1.3)	39 (2.8)
Maternal infections and infestations^h^	7 (4.7)^i^	162 (11.6)
Concomitant medications, *n*/*N* (%)^j^		
Corticosteroids	47/93 (50.5)^k^	267/786 (34.0)
NSAIDs	19/93 (20.4)	117/786 (14.9)
Methotrexate	24/93 (25.8)	80/786 (10.2)
Leflunomide	0	3/786 (0.4)
Azathioprine	0	78/786 (9.9)
Sulfasalazine	18/93 (19.4)	105/786 (13.4)
Opioid analgesics	0	68/786 (8.7)

Abbreviations: CZP, certolizumab pegol; NIS, non‐interventional study; NSAID, non‐steroidal anti‐inflammatory drug; PT, preferred term; SD, standard deviation; SOC, system organ class.

^a^ Clowse M, et al. Ther Adv Musculoskel Dis 2022;14:1–18.

^b^ The higher number of outcomes compared with pregnancies in the global population was due to a number of non‐singleton births.

^c^ All percentages were calculated using the number of pregnancies with known outcomes as the denominator.

^d^ First trimester was defined as the period up to 13 weeks and 6 days' gestation.

^e^ Other was defined as second trimester only, third trimester only, or both second and third trimesters.

^f^ The number of hypertensive disease of pregnancy episodes is based on reported events of hypertension among prospective pregnancies with known outcomes.

^g^ PT: Gestational hypertension.

^h^ SOC: Infections and infestations.

^i^ The most frequent maternal infections and infestations were upper respiratory tract (3.4% of 148 pregnancies), viral (1.4%), skin (0.7%), and lower respiratory tract (0.7%).

^j^ All percentages were calculated using the number of pregnancies with known outcomes, with concomitant medication, as the denominator. Percentages reported are for any use during pregnancy in patients with available information on concomitant medication intake. Due to the sparsity of the information, dose and time of exposure are not included.

^k^ Systemic corticosteroids included prednisolone (36.5% of 148 pregnancies) and methylprednisolone (3.4%).

The mean maternal age was 34.3 years, with approximately half (76/149; 51.0%) of all Japanese women aged ≥ 18 to ≤ 35 years and 60/149 (40.3%) aged > 35 years. Missing ages were reported in 13/149 (8.7%) patients (Table 1). All patients reported singleton births. With regards to the period of CZP exposure, 111/149 (74.5%) and 53/149 (35.6%) were treated with CZP at least during the first trimester and in all trimesters, respectively. Only one pregnancy case involved CZP exposure solely during preconception (Table 1). Hypertensive disease of pregnancy as well as maternal infections and infestations (upper respiratory tract, lower respiratory tract, skin, and viral) were recorded in 2/149 (1.3%) and 7/149 (4.7%) pregnancies, respectively (Table 1). Among 93 patients who received concomitant medications, the drugs used comprised corticosteroids (47/93; 50.5%), methotrexate (24/93; 25.8%), NSAIDs (19/93; 20.4%), and sulfasalazine (18/93; 19.4%) (Table 1). Maternal demographics data previously reported in the global study are included in Table 1 for context [27].

#### 3.2.2 Pregnancy Outcomes

**FIGURE 2:**
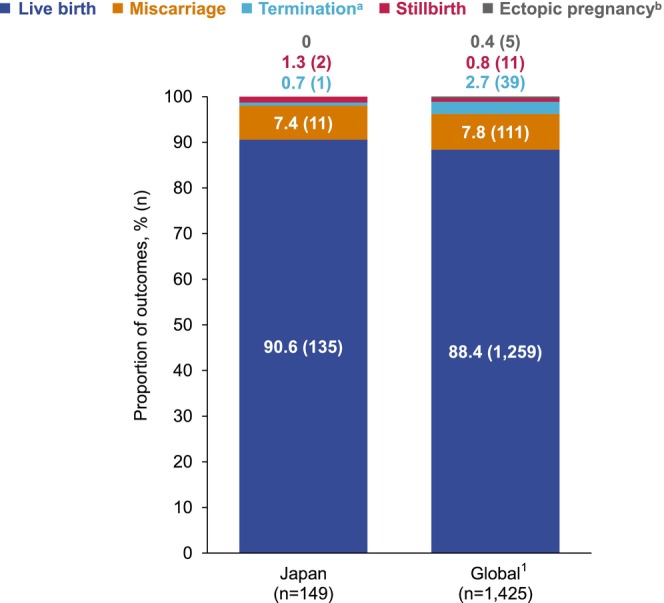
Known pregnancy outcomes of prospectively reported pregnancies with maternal CZP exposure. Data are reported as % (*n*). ^a^Terminations included elective abortions and there were no medically indicated abortions reported; ^b^There were no ectopic pregnancies reported in CZP‐exposed pregnancies in Japanese women. 1. Clowse M, et al. Ther Adv Musculoskel Dis 2022;14:1–18. CZP: Certolizumab pegol.

Of the 149 prospective pregnancies, live births were the most common outcome (135/149; 90.6%). Abortions were recorded in 12/149 (8.1%) cases, which were either miscarriages (11/149; 7.4%) or elective termination (1/149; 0.7%). Of four miscarriages where the gestational age at the time of outcome recording was known, the median gestational age was 9 weeks. Stillbirths accounted for 2/149 (1.3%) outcomes (both cases involved mothers with RA and neither involved anomalies or malformations). Both stillbirths had unknown gestational ages at the time the outcome was recorded. No ectopic pregnancies were reported (Figure 2). Preterm delivery was recorded in 3/135 (2.2%) live birth cases and 2/44 (4.5%) live birth cases where maternal corticosteroid intake was involved (Table 2). Low birth weight was observed in 10/135 (7.4%) infants (Table 2).

**TABLE 2:** Adverse outcomes and congenital anomalies in prospectively reported pregnancies with known pregnancy outcome and maternal CZP exposure.

	Pregnancies with known outcomes	
	Japan	Global^a^
Number of pregnancies	149	1392
Number of outcomes	149	1425^b^
Preterm delivery based on live births, *n*/*N* (%)	3/135 (2.2)	124/1259 (9.8)
With corticosteroid	2/44 (4.5)	44/247 (17.8)
Low birth weight,^c^ *n*/*N* (%)	10/135 (7.4)	101/1259 (8.0)
Congenital anomaly based on all known outcomes, *n*/*N* (%)	1/149 (0.7)	35/1425 (2.5)
Congenital anomaly based on live births, *n*/*N* (%)	1/135 (0.7)	30/1259 (2.4)

Abbreviation: CZP, certolizumab pegol.

^a^ Clowse M, et al. Ther Adv Musculoskel Dis 2022;14:1–18.

^b^ The higher number of outcomes compared with pregnancies in the global population is due to a number of non‐singleton births.

^c^ Gestational age and birth weight were not recorded.

#### 3.2.3 Congenital Anomalies

Among the CZP‐exposed prospective pregnancies with known outcomes, a single case of congenital anomaly (1/149; 0.7%) was reported in a live‐born infant (Table 2). This was determined to be an accessory auricle which was categorized as minor according to the MACDP and EUROCAT criteria [31, 32]. The mother was reported to have had exposure to CZP at preconception and during all trimesters. Among 1425 pregnancies with known outcomes and maternal CZP exposure in the global study, there were 35 (2.5%) cases of congenital anomalies [27]. Similar proportions of congenital anomaly cases were observed among all live births in this Japanese analysis (1/135; 0.7%) and in the global study (30/1259; 2.4%).

Pregnancy outcomes data from the global study are included in Table 2 and Figure 2 for context [27].

## 4 Discussion

In our analysis, pregnancy data from the CZP Pharmacovigilance safety database were used for the first time to assess known pregnancy outcomes with maternal CZP exposure in the Japanese population. A total of 149 prospectively reported pregnancies were analyzed. Approximately 91% of prospective pregnancies resulted in live births, with the remainder of pregnancy outcomes comprising abortions (miscarriage: 7.4%; elective: 0.7%) and stillbirths (1.3%). Only one case of minor congenital anomaly and no major congenital anomalies were reported in this analysis, suggesting no discernible pattern between maternal CZP exposure and congenital anomalies or malformations.

Overall, the results regarding pregnancy and adverse birth outcomes in Japanese women were consistent with previously published global data from the CZP Pharmacovigilance safety database which reported prospective pregnancy outcomes using the cut‐off dates of March 6, 2017 (528 pregnancies) and November 1, 2020 (1392 pregnancies) [27, 28]. This suggests that CZP therapy may be used by pregnant Japanese women or those planning pregnancy to manage CID before, during, and after pregnancy without increasing their risk of adverse pregnancy outcomes. Given the consistency between the Japanese and global safety data regardless of demographic variations, our findings may be generalizable to the wider Asian population. Future research to examine cross‐population consistencies in safety should be considered to enhance the understanding of the impact of CZP treatment on pregnancy and live birth outcomes in the broader Asian region.

These results were aligned with the respective trends in pregnancy outcomes of the general population in Japan [33, 34, 35, 36, 37]. Rates of adverse outcomes among live‐born infants of this study were comparable with those observed in the general population, namely preterm delivery (5.6%) and low birth weight (9.4%) [38, 39]. The population rate for major congenital anomalies ranged 1.3%–2.9% in Japan [35, 40, 41]. In this study, only a single case of minor anomaly (accessory auricle) was recorded, which would not have been included in the population‐based analyses that only considered cases with major anomalies. Of note, accessory auricles generally have an incidence of 5–10 per 1000 live births and most cases do not require surgical interventions unless for cosmetic purposes [42, 43]. Comparisons between findings from the safety database analyses and data on the prevalence of pregnancy and live birth outcomes in the Japanese general population should nonetheless be interpreted with caution due to inherent limitations. These include differences in criteria used to classify major anomalies or malformations, variations in study design, and the times at which the studies were conducted.

Studies reporting adverse pregnancy outcomes specifically in Japanese women exposed to biologics were notably sparse. In two Japanese studies which separately examined the impact of tocilizumab and adalimumab on pregnancies, no congenital anomalies were detected. However, the authors noted that the rates of preterm delivery and low birth weight from tocilizumab‐exposed pregnancies resulting in live‐born infants (*n* = 36) were 5.6% and 13.9%, respectively, and for adalimumab (*n* = 45), 4.4% and 11.1%, respectively. Both investigations also reported that among all pregnancies with known outcomes (tocilizumab, *n* = 50; adalimumab, *n* = 53), miscarriage rates were 18.0% and 9.4%, respectively [44, 45]. It should be considered that the patient populations in these prior Japanese studies may have demographics and characteristics that differ from those in our study. Comparisons between our findings and previously published data on these other biologics may therefore be confounded by several variables and should be examined carefully.

Adequate control of CID remains the key contributor to reducing the risks of adverse pregnancy outcomes [46]. Treatments which demonstrate efficacy and safety across different trimesters of pregnancy would thus be advantageous. Previously published pharmacovigilance data suggest that CZP exposure in the first trimester (when organogenesis occurs) does not increase the risk of adverse pregnancy outcomes and congenital anomalies [47]. Additionally, the minimal level of placental transfer of CZP from mother to infant results in a lower likelihood of in utero fetal exposure in all trimesters, especially the second and third trimesters when transplacental IgG transport is most active [23]. In this study, no meaningful differences were observed in pregnancy and adverse birth outcomes between pregnancies exposed to CZP at least during the first trimester, during all trimesters, and other timings. As only one mother reported CZP exposure exclusively at preconception, limited conclusions could be made about the impact of CZP administered during this stage on pregnancy outcomes.

Regarding concomitant medication intake to manage CIDs, while it was expected that there would be some patients taking methotrexate preconception (defined in the pharmacovigilance database as 70 days prior to conception), the proportion of pregnancies identified as exposed to the drug was markedly high (24/93 [25.8%] pregnancies with concomitant medications). However, further information including the exact timing of exposure and interruption (postulated as cessation of methotrexate immediately upon discovery of pregnancy or due to induced abortion) were not recorded in the database or elucidated at the time of the analysis. Hence, the effect of methotrexate on pregnancy outcomes could not be ascertained in our investigation.

A potential drawback of this study was the vast majority of prospective pregnancies which comprised patients who reported RA (*n* = 145) as an indication for CZP treatment. The few remaining pregnancies comprised one case involving PSO and three with missing diagnosis data. Whereas in the global study, the distribution of indications for CZP treatment included mostly RA (*n* = 643), followed by Crohn's disease (*n* = 293), axSpA (*n* = 215), PsA (*n* = 113), PSO (*n* = 61), and other CIDs (*n* = 52) [27]. The difference in distributions may contribute to challenges in comparing results between the Japanese and global analyses. However, it was noted that the reported indications may not contribute as substantially to teratogenicity and adverse pregnancy outcomes compared with uncontrolled disease activity (regardless of indication) and potentially, the kinds of therapies administered [48, 49].

Other limitations of this study were similar to those previously reported in the global study [27]. Firstly, a passive reporting system has the propensity for underreporting and missing key data such as disease activity during pregnancy and history of childbirth with adverse outcomes. The sparsity of information on concomitant medication intake was also a barrier to the inclusion of dose and timing of exposure data in this analysis. Consequently, limited conclusions could be drawn about the influence of concomitant medications on CZP‐exposed pregnancies and the independent impact of disease activity on pregnancy outcomes. The lack of a control group comprising pregnancies in women with CID but not exposed to CZP had also prevented further analysis of outcomes and risk assessment. Additionally, the absence of a control group may have limited the ability to establish associations between CZP intervention and the trends observed in this study. However, it is acknowledged that the inclusion of a control group may not always be possible for studies involving pregnancy. This is because unexposed pregnancies are not included in pharmacovigilance datasets, and risk assessments of medicinal products on human reproduction typically rely on evaluations of pregnancies in patients who are already exposed [50]. Lastly, given the small sample size of prospective pregnancies in Japanese women involved in this study, the meaningfulness and generalizability of our findings to real‐world clinical practice may be limited.

The large proportion of CZP‐exposed pregnancy cases with known outcomes in the Japanese cohort which were sourced from spontaneous reports underscores the commitment of physicians toward active outcomes reporting in Japan. Such contributions enabled these analyses and similar reporting attitudes should be encouraged in the wider community of healthcare professionals to empower robust pharmacovigilance assessments in different geographies.

## 5 Conclusion

**FIGURE 3:**
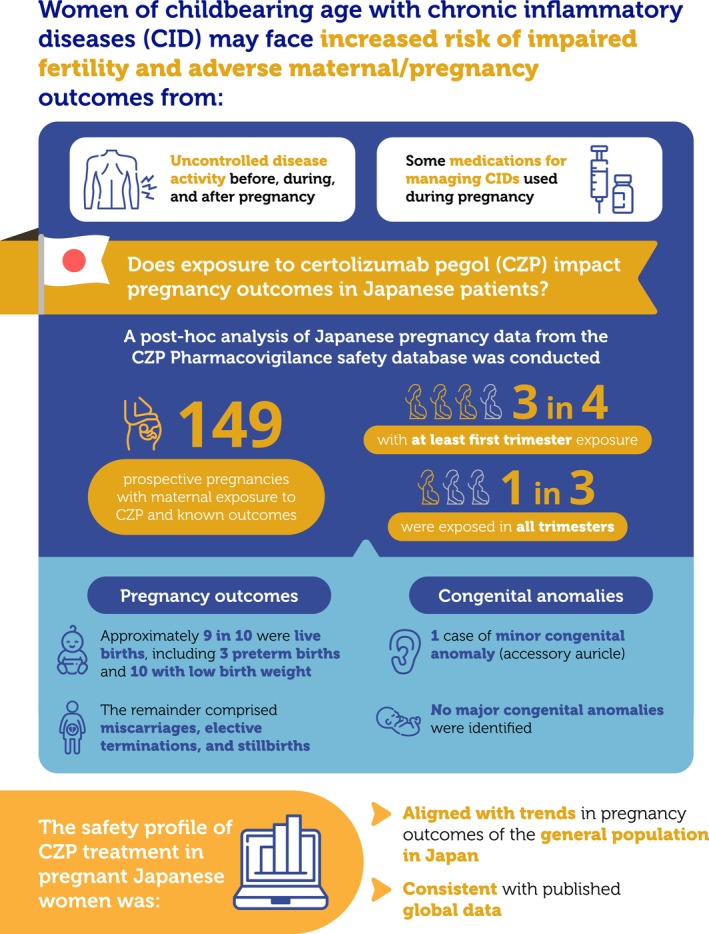
Graphical abstract. CID, Chronic inflammatory disease; CZP, Certolizumab pegol.

In summary, our study represents the first time prospective pregnancy cases from the CZP Pharmacovigilance safety database were used to evaluate the impact of maternal CZP exposure on known pregnancy outcomes in Japanese women with CID. This review reflects the consistency of the safety profile of CZP treatment in pregnant Japanese women with previously published data from the global study. A graphical abstract which summarizes this manuscript is provided in Figure 3.

## Author Contributions

Substantial contributions to study conception and design: Mikako Goto, Shigeru Saito, Angela E. Scheuerle, Shinya Yasuda, Niamh Houston, Thomas Kumke, Bernard Lauwerys, Atsuko Murashima. Substantial contributions to analysis and interpretation of the data: Mikako Goto, Shigeru Saito, Angela E. Scheuerle, Shinya Yasuda, Niamh Houston, Thomas Kumke, Bernard Lauwerys, Atsuko Murashima. Drafting the article or revising it critically for important intellectual content: Mikako Goto, Shigeru Saito, Angela E. Scheuerle, Shinya Yasuda, Niamh Houston, Thomas Kumke, Bernard Lauwerys, Atsuko Murashima. Final approval of the version of the article to be published: Mikako Goto, Shigeru Saito, Angela E. Scheuerle, Shinya Yasuda, Niamh Houston, Thomas Kumke, Bernard Lauwerys, Atsuko Murashima.

## Conflicts of Interest

**M.G.:** None. **S.S.:** Received speaker fees from UCB and Astellas Pharma Inc. **A.E.S.:** Received consultancy fees from Antiretroviral Pregnancy Registry, Harmony Biosciences, IQVIA, PPD, Sanofi‐Genzyme, Syneos, UCB, and ViiV. **S.Y.:** Employee of UCB. **N.H**., **T.K**., and **B.L.:** Employees and shareholders of UCB. **A.M.:** Received research grants from Asahi Kasei Pharma Corporation and Chugai Pharmaceutical Co. Ltd. and speaking fees from Asahi Kasei Pharma Corporation, Astellas Pharma Inc., Chugai Pharmaceutical Co. Ltd., and UCB.

## Data Availability

Data from non‐interventional studies are outside of UCB's data sharing policy.
